# Enhanced Deep Ultraviolet Photoresponse in Ga doped ZnMgO Thin Film

**DOI:** 10.3390/mi13071140

**Published:** 2022-07-19

**Authors:** Mao Ye, Dongbo Wang, Shujie Jiao, Lang Chen

**Affiliations:** 1 Department of Physics, Southern University of Science and Technology, Shenzhen 518055, China; yem@sustech.edu.cn; 2 Department of Optoelectronic Information Science, School of Materials Science and Engineering, Harbin Institute of Technology, Harbin 150001, China; shujiejiao@hit.edu.cn

**Keywords:** deep-UV, ZnMgO, Ga dope, Ga_2_O_3_, magnetron sputtering

## Abstract

High Mg content (60%) ZnMgO samples with and without Ga dope were grown by an RF magnetron sputtering system. The effect of Ga dope on the ZnMgO sample and the respective ultraviolet photodetectors (UVPD) device’s performance were carefully studied by various experimental methods. The investigations of the structure and optical properties of the ZnMgO sample established that the Ga doped sample has a better crystal quality and larger band gap (5.54 eV). The current-voltage characteristics indicate that both the photocurrent and dark current were enhanced after Ga dope. Under 12 V bias, the undoped UVPD show two spectral response peaks at 244 nm and 271 nm with a responsivity of 1.9 A/W and 0.38 A/W, respectively. While the Ga doped UVPD showed only one response peak at 241 nm and the deep UV responsibility up to 8.9 A/W;, as the bias increased from 12 V to 60 V, the responsiveness raised to 52 A/W, with a signal to noise ratio (241 nm/700 nm) as high as 10^5^. Combining the results of XRD, PL spectrum and XPS, the enhanced ultraviolet photoresponse of the Ga dope device contributed to improving the crystal quality and “dopant-defect pairing effect” caused by Ga doping, which led to a considerable reduction in the number of ionized impurities in the scatting centers, and enhanced the carrier’s mobility. Our work demonstrates that even a high Mg content ZnMgO can exhibit enhanced UV performance after a Ga dope due to the dopant-defect pairing effect, which confirmed the advantage of the use of ZnMgO in the deep-UV region.

## 1. Introduction

Ultraviolet photodetectors (UVPDs) have wide applications in both civil and military areas, such as space probing, chemical, and biological detection, missile early-warning, flame detecting, biological research, and optical communications [1,2,3,4,5]. Among those semiconductor materials that have a wide band gap (GaN, SiC, diamond, ZnS), ZnO stands out as a promising candidate for UV detection due to its large exciton binding energy (60 meV); direct wide band gap (3.4 eV); environmentally friendly properties; and chemical and thermal stability [6,7,8,9]. Furthermore, the band gap of ZnO can be tailored by forming alloys with other oxides, such as MgO [10], CdO [11], and BeO [12]. In particular, the ZnMgO alloys can enlarge the band gap of ZnO from 3.4 to 7.8 eV, which allows ZnO to work in the deep-UV region and fabricate ZnMgO UVPDs with different cutoff wavelengths by adjusting the Mg content [13,14,15,16]. However, as is well known, ZnO crystallizes and is stabilized in the hexagonal wurtzite lattice, whereas MgO is stabilized in the cubic rock salt lattice. As the Mg content increases, the crystalline structure of ZnMgO ternary alloy will transform through the wurtzite phase (low Mg content), mixed-phase (both hexagonal and cubic phase), and cubic phase (high Mg content) [14,17]. UVPDs based on cubic rock-salt ZnMgO (c-ZnMgO), wurtzite ZnMgO (w-ZnMgO), and mixed-phase ZnMgO (m-ZnMgO) have been reported [18,19,20]. The performance of ZnMgO UVPDs strongly relies on the Mg content; when the Mg concentration is above 33%, ZnMgO ternary alloys are segregated into mixed-phase, and the crystal quality normally decreases with the increase of Mg content and misfit dislocations [21,22]. Furthermore, the conductivities of ZnMgO films usually decrease when the Mg content rises [23,24]. For instance, Matsubara reported that the resistivity of MgZnO varied between 3 × 10^−4^ Ω cm and 1 × 10^−3^ Ω cm as the band gap was increased from 3.5 eV to 3.97 eV [24]. Both of them seriously restricted the development of ZnMgO deep-UV optoelectronic devices. Until now, the performance of c-ZnMgO UVPDs (high Mg content) is still worse than “that of w-ZnMgO” (low Mg content) [25].

The performance of ZnMgO UVPDs strongly relies on the Mg content; when the Mg concentration is above 33%, ZnMgO ternary alloys are segregated into mixed-phase, and the crystal quality normally decreases with the increase in Mg content and misfit dislocations [21,22]. Furthermore, the conductivities of ZnMgO films usually decrease when the Mg content rises [23,24]. For instance, Matsubara reported that the resistivity of MgZnO varied between 3 × 10^−4^ Ω cm and 1 × 10^−3^ Ω cm as the band gap was increased from 3.5 eV to 3.97 eV [24]. Both of them seriously restricted the development of ZnMgO deep-UV optoelectronic devices. Until now, the performance of c-ZnMgO UVPDs (high Mg content) has been still worse than “that of w-ZnMgO” (low Mg content) [19].

To realize high-performance deep-UV optoelectronic devices, great efforts have been devoted to studying doped ZnMgO [25], such as Dy [26], Ag [27], Al [28], Ga [29], and As [30]. Among these elements, Ga is the most effective n-type dopant in ZnO ascribing to the covalent bond length of Ga-O (1.92 Å) is similar to that of Zn-O (1.97 Å). Meanwhile, the different growth temperatures of ZnO (950 °C) and Ga_2_O_3_ (1100 °C) in a vapor–solid progress is conducive to regulating the Ga-doping component [31]. Recently, the first-principle calculation has shown that Ga dope can enhance the electron mobility of ZnO through the dopant-defect pairing effect [32]. 

Unfortunately, up to now, high Mg content (>50%) Ga doped ZnMgO UVPDs and their optical response performance have been full of challenges and seldom reported. 

In this work, we explore the effect of Ga dope on the photoresponse properties of ZnMgO deep-UVPDs. ZnMgO films with and without Ga dope were grown and their respective UVPDs device performances were carefully studied. The Ga doped ZnMgO has a better crystal quality and only one band gap (5.54 eV) compared with the undoped ZnMgO. Enhanced deep-UV photoresponse was found in the Ga-doped UVPDS with a cut-off wavelength of about 241 nm. The mechanism of the enhanced UV photoresponse in Ga-doped ZnMgO alloys was discussed in detail through careful inspections of photoresponse properties, combined with the result of XPS and photoluminescence.

## 2. Materials and Methods

### 2.1. Growth of Sample

ZnMgO samples with and without Ga dope were all deposited on quartz substrates through an RF magnetron sputtering system [33] in a vacuum (4.9 × 10^−3^ Torr) chamber with the same growth condition. Before deposition, the substrates were cleaned with ultrasonic vibration in ethanol and high purity water.

The flow rate of Ar and O_2_ was 8:32. And the working pressure was 0.5 Pa. For all the samples, the sputtering power was kept at 70 W, and the sputtering time was 120 min. The distance between the sample and target was 10 cm.

The ZnMgO target was prepared by the sintering of a mixture of 99.99% pure MgO and ZnO powders. The target contained 60 at% Mg, and 40 at% Zn. While Ga: ZnMgO target was also prepared by a sintering mixture of 99.99% pure MgO, ZnO, and Ga_2_O_3_ powders. The target contained 57 at% Mg, 40 at% Zn, and 3 at% Ga.

### 2.2. Fabrication of the UVPDs

**Figure 1:**
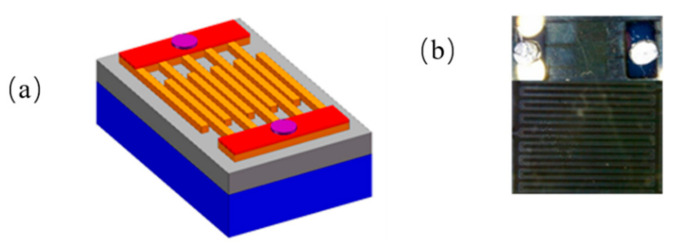
(**a**) The diagrammatic structure of the UVPDs, (**b**) The schematic full view of the interdigital metal electrodes of the UVPDs.

The metal–semiconductor–metal (MSM) deep-UVPDs were fabricated using the standard semiconductor fabrication techniques, which is a photolithography process employed to pattern the symmetric metal electrode pairs [34,35,36]. The diagrammatic structure of the PEC UVPD is shown in Figure 1a. The interdigital metal electrodes, which were defined on a 200 nm Au layer by the conventional UV photolithography and lift-off procedure, are 500 μm long and 5 μm wide, with a 2 μm gap. Figure 1b shows the schematic full view of the interdigital metal electrodes of the UVPDs, in which the yellow and white parts are the electrode and sample, respectively. There are 80 fingers in our interdigital structure, 40 up and 40 down [37].

### 2.3. Characterization

All the samples were characterized by using X-ray diffraction (XRD) (Panalytical Empyrean, Eindhoven, The Netherlands), X-ray photoelectron spectroscopy (XPS) (ESCALAB 250Xi, ThermoFisher Scientific, Waltham, MA, USA), Energy Dispersive Spectrometer (EDS) (Quanta 200FEG); photoluminescence (PL) characterizations were recorded in CCD spectroscopy at a fixed excitation intensity by a He-Cd laser line 325 nm (HORIBA, LabRAM HR Evolution, Longjumeau, France). LAMBDA UV/Vis Spectrophotometers 850 measured the optical transmittance and reflectance characterizations. Responsivity measurements were carried out with a spectrograph (Zolix, Omni-3007, Beijing, China), photodetector (Zolix, QE-B3-UV, Beijing, china), and a digital data acquisition system (FS-Pro, KEITHLEY/2400, SW Karl Braun Drive, Beaverton, OR, USA). The light source was provided by a 200 W UV-enhanced Xe lamp (Zolix, Beijing, China).

## 3. Results and Discussion

**Figure 2:**
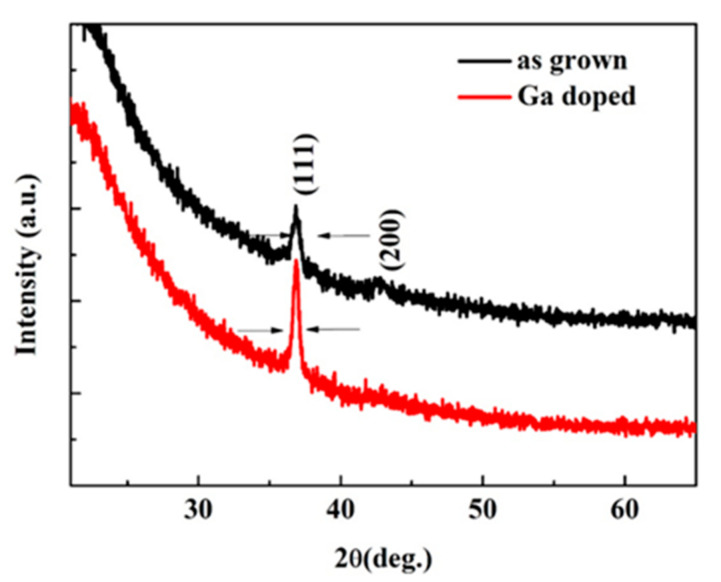
The XRD pattern of the ZnMgO sample with and without Ga dope.

The XRD pattern of the samples is shown in Figure 2. The diffraction peaks at about 36.84° originate from c-ZnMgO (111) [38], and the peak located at 42.45° is from c-ZnMgO (200) [21]. 

For the Ga dope ZnMgO sample, there is only one peak observed, implying that the phase separation has been suppressed. Furthermore, after Ga dope, the intensity of c-ZnMgO (111) increases, which indicates that Ga dope ZnMgO is crystallized with (111)-c ZnMgO prefer orientation. Besides, the (111) c-ZnMgO shifts to 36.95°. According to Bragg’s equation [39]

2*dsinθ* = *nλ* (1)

(2) $$d_{hkl}=a/\sqrt{h^{2}+k^{2}+l^{2}}$$

where *θ* is the diffraction angle, *d* is the Miller indices, and *a* is the lattice constant. According to the formulas (1) and (2), the diffraction angle *θ* is in inverse proportion to the lattice constant *a*, since the lattice constant of ZnO is larger than the MgO [40], so the diffraction peaks of the c-ZnMgO shifting to the high angle side can be ascribed to the decrease of the lattice constant due to the increase in Mg incorporation. In addition, the full width at half-maximum (FWHM) of the (111) XRD rocking curves was obtained by fitting the XRD diffraction peak curve. The FWHM of the (111) XRD rocking curves are 0.62° and 0.44° for the as-grown and Ga doped samples, respectively. This result implies that the crystal quality of ZnMgO improves after Ga dope. 

The composition of ZnMgO is measured by the EDS, XPS as summarized in Table 1. More information about EDS and XPS is shown in the support information.

**Figure 3:**
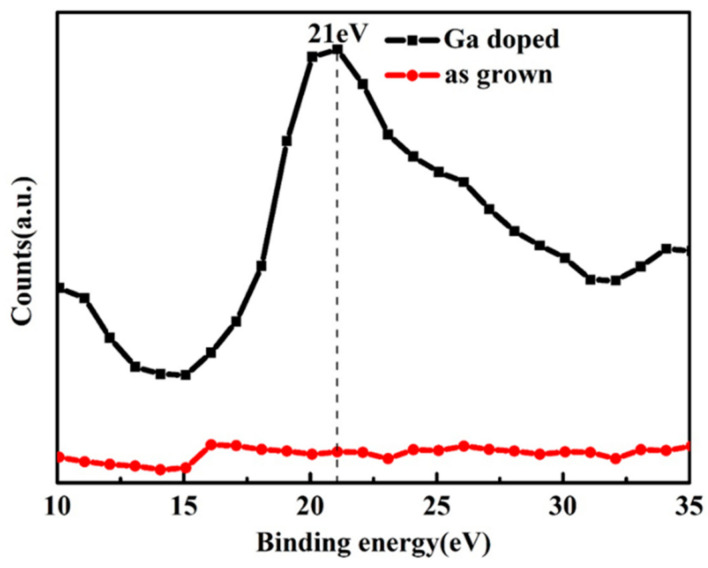
XPS survey spectrum of the ZnMgO sample with and without Ga dope.

The XPS spectra is used to measure the Ga content of the sample in Figure 3. The peak located at 21 eV is ascribed to Ga 3d [41], which reflects the Ga-O chemical bonding in Ga_2_O_3_

**Figure 4:**
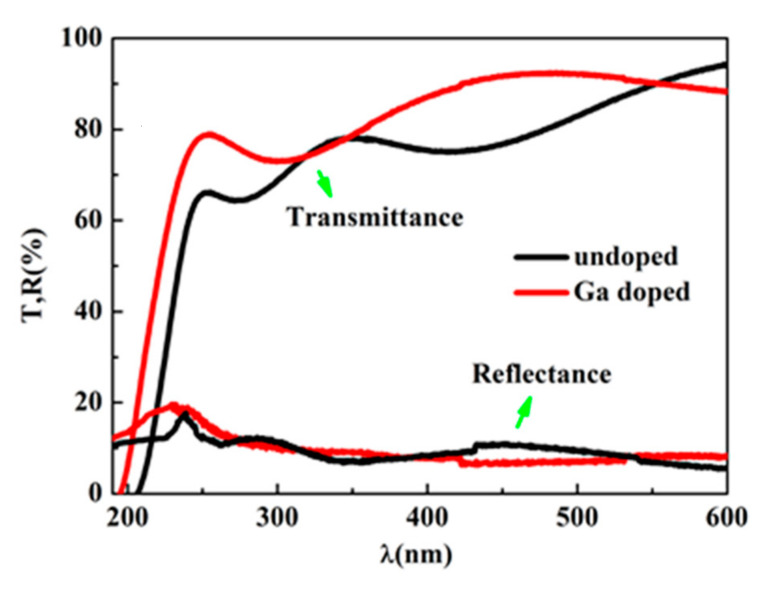
Optical properties of ZnMgO samples with and without Ga dope.

Optical transmittance spectra and reflectance spectra of the samples are shown in Figure 4. It can be seen that both samples have over 70% transmittance in the UV and visible region of 250–600 nm. After doping with Ga, the transmittance edge shifted to a shorter wavelength. In addition, after doping with Ga, the transmittance of ZnMgO was enhanced. 

**Figure 5:**
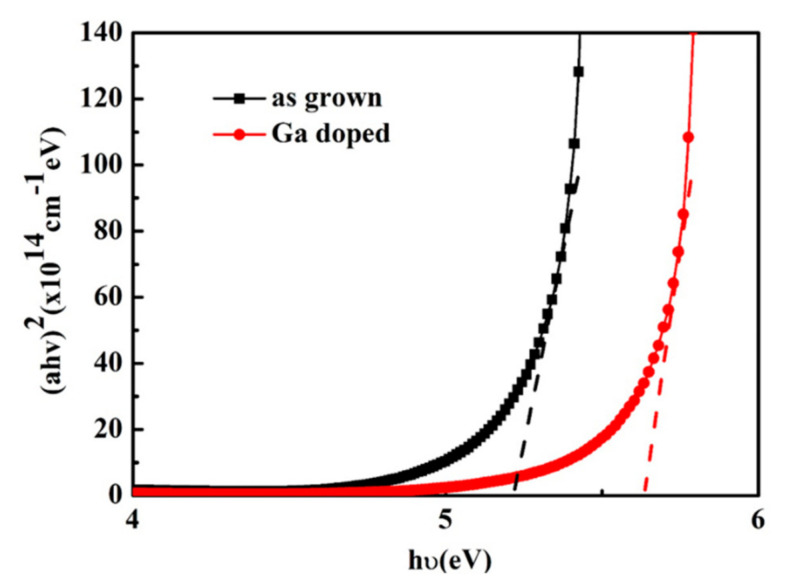
The curve of (*ahv*)^2^ versus *hv* for ZnMgO samples.

The relation between the transmittance (*T*) and absorption coefficients (*a*) can be expressed as [42]:

*a* = (*InT* − 1)/*d* (3)

(*ahv*)^2^ = *A*(*hv* − *E_g_*) (4)

where *A* is a constant, *d* is the thickness of the films, *E_g_* is the band gap of the sample. The band gap of the ZnMgO samples can be estimated from the extrapolation approach shown in Figure 5.

The band gaps are 5.54 eV and 5.20 eV for the Ga doped and undoped ZnMgO films, respectively. After Ga dope the band gap of ZnMgO increases, which indicates an increase in Mg content. The result is consistent with the conclusion of XRD and XPS.

**Figure 6:**
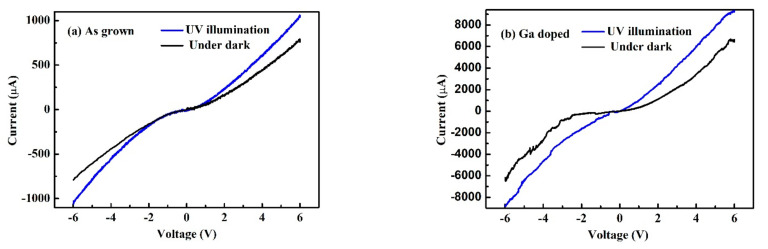
The current-voltage curve of the ZnMgO sample. (**a**) as grown and (**b**) with Ga dope.

The current-voltage characteristics of the ZnMgO sample with and without Ga dope are measured under dark and UV illumination conditions, respectively. In Figure 6a, under UV illumination, at 5 V bias, the current for undoped and doped ZnMgO UVPD was 0.82 mA and 5.2 mA, respectively. While under dark conditions, the dark current for undoped and doped ZnMgO was 0.62 mA and 3.4 mA, respectively. The results I–V indicate that Ga dope can effectively enhance the current of UVPD.

**Figure 7:**
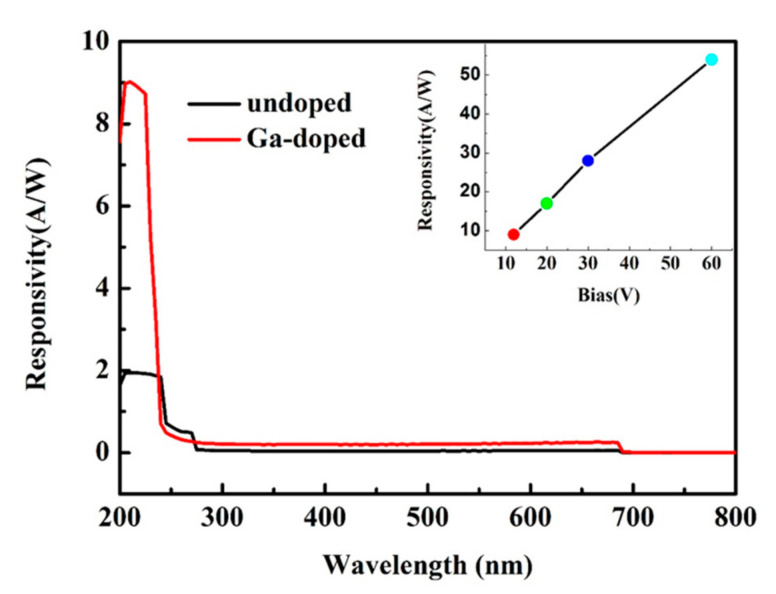
Spectral response of the ZnMgO sample with and without Ga dope.

**Figure 8:**
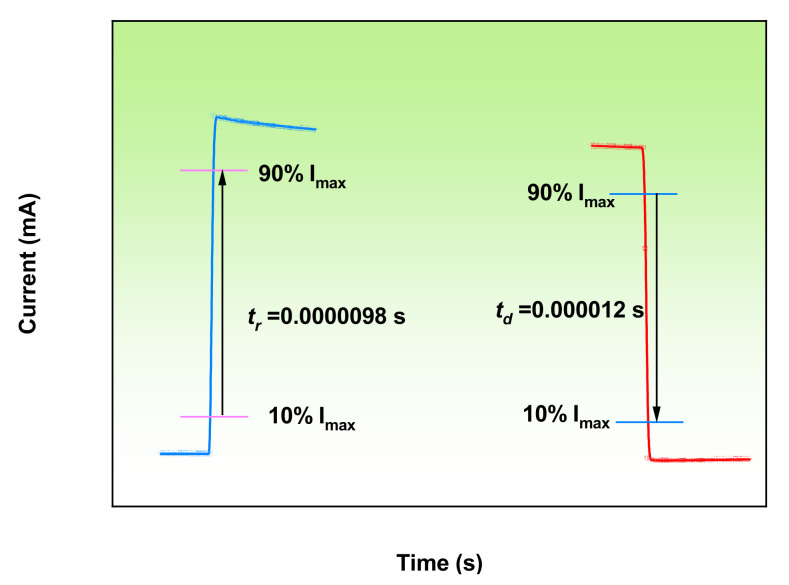
Response time of the detector.

To test the spectral responsivity of the photodetector, the wavelength selective property was measured in terms of the current signal in the range of 200–800 nm at a 12 V bias, and the results are shown in Figure 7. Photoresponsivity is an important property for photodetector performance, which can directly affect the final device spectral response. For the sample without Ga dope, the UVPD has two obvious spectral responses corresponding to a cutoff wavelength of 271 nm and 244 nm, with a responsivity of 1.32 A/W and 0.38 A/W, respectively. While the Ga doped UVPD, the spectral response at 271 nm disappeared and the deep UV spectral response shifted to 241 nm with photoresponsivity up to 8.9 A/W, a signal-to-noise ratio (241 nm/700 nm) as high as 10^5^, nearly five times that of the undoped one. The inserted figure shows the response varying with bias, as the bias increased from 12 V to 60 V, the responsiveness rose to 52 A/W. The response time before and after Ga doping is 300 ms and 55 ms, respectively. In order to improve the response time, Ga doped samples and Bi_2_Se_3_ were used to prepare the heterojunction, and the response time of the detector reached 98 μs (Figure 8).

The spectral response results indicate that Ga dope could make the high Mg content (60%) ZnMgO UVPDs obtain a single cut-off wavelength response in the deep ultraviolet region, and enlarge the cut-off wavelength (band gap) of the ZnMgO. This is consistent with the XRD result that Ga dope can suppress the phase separation and improve the crystal quality of the high-Mg-content ZnMgO work in deep-UV regions.

In order to further explore the mechanism of the enhanced spectral response, the cut-off wavelength, carrier concentration, and electron mobility of the ZnMgO samples were measured by Hall effect measurement and listed in Table 2. The cut-off wavelength was obtained by measuring the spectral response cut-off band edge position.

It is worth noting that the carrier concentrates and electron mobility of the ZnMgO sample were both enhanced after Ga dope. Usually, due to grain barrier limited transport [43,44], the electron mobility decreases with the increase of the carrier concentration.

The situation here is similar to the dopant-defect pairing effect calculated through density functional theory (DFT) in Al, Ga doped ZnO [32,45,46].

As they are calculated, Zn vacancies (V_Zn_) lead to reduced electron mobility in the ZnO which act as the additional ionized impurity scattering centers [47]. Due to oppositely charging, defects tend to form pairs and complexes [48]; the V_zn_ and intentional Ga_Zn_^+^ dopant are finally formed singly charged (Ga_Zn_-V_Zn_)^−^ and the charge-neutral (2Ga_Zn_-V_Zn_)^−^ defect clusters [49,50], lead to reducing considerably the number of ionized impurities in the scatting centers and enhance the mobility.

**Figure 9:**
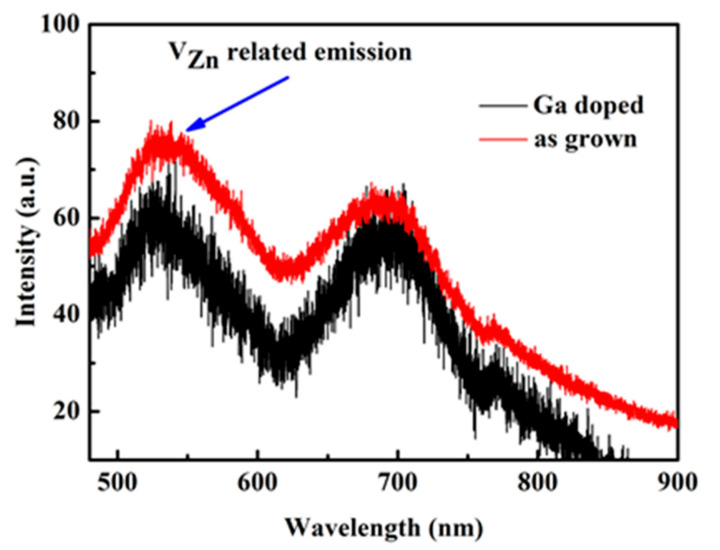
PL spectra of the ZnMgO sample at room temperature.

The PL spectrum is used to characterize the change in V_Zn_ before and after Ga dope (Figure 9). It was observed that there are two obvious emission peaks in the visible region. According to the previous reports, the emission located at 530 nm belongs to V_Zn_ [51,52] and the other emission peaks are ascribed to the excess oxygen on the ZnO surface [53,54,55]. As can be seen from Figure 9, the V_zn_ related emission decreases after Ga doping, which may reduce the number of V_zn_.

**Figure 10:**
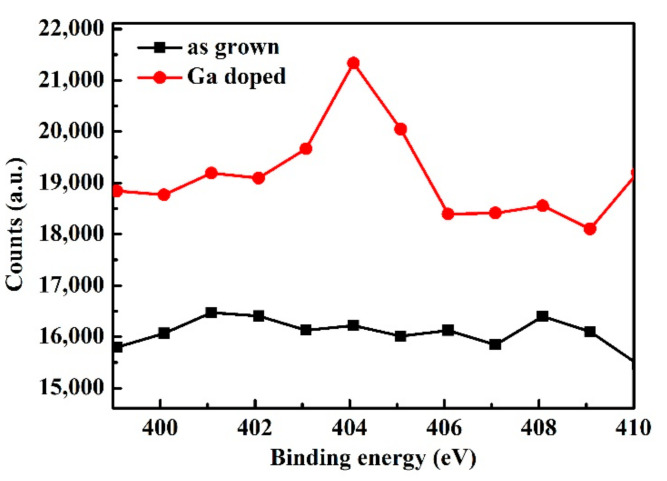
XPS survey spectrum of the ZnMgO samples.

Furthermore, the binding energy of (Ga_Zn_-V_Zn_)^−^ calculated by the DFT method is 3.05 eV (*λ* = 1.24/*E_g_*, 406 nm) [32]. Meanwhile, in the XPS spectrum (Figure 10), the Ga doped sample emerges at an obvious peak at 409 nm, which is similar to the calculated value. These results indicated that (Ga_Zn_-V_Zn_)^−^ exist in ZnMgO after doping with Ga.

Therefore, it is reasonable to conclude that Ga dope in high Mg content ZnMgO films would enhance ultraviolet photoresponse due to the dopant-defect pairing effect.

Combined with the above results, Ga’s role in influencing the performance of the device was elaborated in detail. Firstly, Ga dope can improve the crystal quality of MgZnO, thereby improvingthe photocurrent of the device. Secondly, in the ZnMgO alloys, the formation of acceptor-like compensating intrinsic defects, such as zinc vacancies (V_Zn_), always increases with the rise of Mg content, which reduces the free electron concentration and decreases the mobility through ionized impurity scattering. After being Ga doped, the V_zn_ and intentional Ga_Zn+_ dopant can finally form singly charged (Ga_Zn_-V_Zn_)^−^ and the charge-neutral (2Ga_Zn_-V_Zn_)^−^ defect clusters, which reduces considerably the number of ionized impurities in the scatting centers, and enhances the carrier’s mobility. An increase in the carrier’s mobility enhances the conductivity of the material and improves the device’s performance.

## 4. Conclusions

In summary, the effect of Ga dope on the ZnMgO films and the respective UVPD-device performance was carefully studied by various experimental methods, and the mechanism of the enhanced spectral response was explored. The Ga doped sample shows a single c-ZnMgO (111) diffraction peak and narrow FWHM, which indicates that Ga dope can improve the crystal quality of ZnMgO. Under the same bias, the photocurrent of UVPD was significantly enhanced after Ga dope. Under 5 V bias, the undoped UVPD showed two spectral response peaks at 244 nm and 271 nm, while the Ga dope UVPD showed only one response peak at 241 nm and enhanced the deep-UV responsibility. Combining the results of XRD, PL spectrum, and XPS, the enhanced ultraviolet photoresponse of the Ga dope device contributedto improving the crystal quality and “dopant-defect pairing effect” caused by Ga doping, which leads to a considerable reduction in the number of ionized impurities in the scatting centers, and enhances the carrier’s mobility. Our work demonstrates that even a high Mg content ZnMgO can exhibit enhanced UV performance after Ga dope, which confirms the advantage of ZnMgO in the deep-UV regions.

## Figures and Tables

**Table 1:** Structure properties of ZnMgO sample.

Sample	Composition (EDS)	Composition (XPS)	FWHM
Ga undope sample	Zn_0.46_Mg_0.54_O	Zn_0.46_Mg_0.54_O	0.62°
Ga dope sample	Zn_0.39_Mg_0.58_Ga_0.03_O	Zn_0.41_Mg_0.57_Ga_0.02_O	0.44°

**Table 2:** The electrical properties of the sample.

Sample	Cut-Off Wave-Length	Carrier Concen-Trate (10^16^ cm^−3^)	Electron Mobility (cm^2^/V∙s)	T
Ga dope sample	241 nm	2.87	6.73	N
Ga undope sample	244 nm	0.19	2.26	N

## Data Availability

The data presented in this study are available on request from the corresponding author.
